# Gombapyrones E and F, New *α*-Pyrone Polyenes Produced by *Streptomyces* sp*.* KMC-002

**DOI:** 10.3390/molecules16053519

**Published:** 2011-04-26

**Authors:** Hyun Bong Park, Hyun Ok Yang, Kang Ro Lee, Hak Cheol Kwon

**Affiliations:** 1 Natural Medicine Center, Korea Institute of Science and Technology (KIST), Gangneung, Gangwon-do 210-340, Korea; Email: 007geeny@hanmail.net (H.B.P.); hoyang@kist.re.kr (H.O.Y.); 2 Natural Products Laboratory, School of Pharmacy, Sungkyunkwan University, Suwon 440-746, Korea; Email: krlee@skku.edu (K.R.L.)

**Keywords:** *Streptomyces*, gombapyrone, *α*-pyrone, polyene

## Abstract

Microorganism-derived polyene polyketides have been shown to display a variety of biological activities and have attracted great interest due to their structurally intriguing chemical diversity. Two new polyenes were isolated from a culture broth of *Streptomyces* sp. KMC-002 obtained from a soil sample in an abandoned mine. The structures of these compounds were determined to be *α*-pyrone-containing polyene analogues through analyses of HRFABMS, UV and NMR data, and were named Gombapyrones E (**1**) and F (**2**). Gombapyrone E (**1**) showed antibacterial activity against *Micrococcus luteus*, *Enterococcus hirae*, *Staphylococcus aureus* and MRSA.

## 1. Introduction

**Figure 1:**
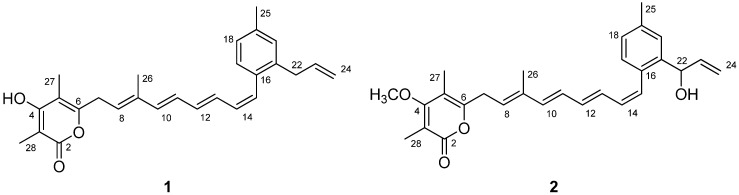
Structures of gombapyrone E (**1**) and F (**2**).

Polyketides represent a highly diverse structural class of natural products found in bacteria, fungi, marine invertebrates, insect and higher plants [1,2,3,4,5,6,7]. From a biochemical point of view, they are thought to be involved in many different types of biological events acting as pheromones, offense and defense materials, pigments and biosynthetic intermediates [8,9,10,11]. Polyketide biosynthesis involves a series of enzyme-catalyzed processes taking place by successive decarboxylative Claisen condensations of activated acetyl-derived starter and malonyl-derived extender units [12,13]. Programmed events, modulated by variety of catalytic domains, endow them with structurally fascinating carbon backbones, ranging from simple pyrone groups to highly complex polyene macrolides [14,15,16,17]. In addition, polyketide natural products have provided many pharmaceutical lead compounds such as polyene-polyol macrocyclic lactones, nystatin and amphotericin B [18,19,20,21,22]. Because of their large structural diversity and varied biological functions, it is an undeniable fact that polyketide natural products are one of the attractive groups for developing useful chemicals as therapeutic approaches or as synthetic challenges [23,24,25]. In our preliminary screening for discovery of new polyketides produced by abandoned mine-derived actinomycetes, two new polyene-polyketides, gombapyrone E (**1**) and F (**2**), were isolated from organic extract of *Streptomyces* sp. KMC-002. The structures of these compounds (Figure 1), which represent new members of the gombapyrone family previously elucidated by Helaly *et al*. [26], were assigned by analyses of 2D-NMR, UV and HRFABMS (High-Resolution Fast Atom Bombardment Mass Spectrometry) data. Previously described gombapyrones possess triene chains attached to an aromatic ring, whereas gombapyrones E (**1**) and F (**2**) isolated from *Streptomyces* sp. KMC-002 are composed of *α*-pyrone and phenyl groups connected through a conjugated tetraene polyketide chain. Although the carbon skeleton of these compounds resembles the previously reported CRP-2504-1a, polyketide-derived tetraene metabolites with *α*-pyrone moieties are extremely rare in Nature [27]. We thus describe here the isolation, structure elucidation and antimicrobial activity of two new *α*-pyrone polyenes isolated from an abandoned mine-derived *Streptomyces* sp.

## 2. Results and Discussion

The sequence analysis of the 16S rRNA gene placed the strain KMC-002 within the genus *Streptomyces* on the basis of the 99.1% similarity with *Streptomyces albireticuli* strain JCM 4116 (AY999748). The cultured *Streptomyces* strain KMC-002 was deposited at the Korean Culture Center of Microorganisms (KCCM 90098).

The strain KMC-002 was isolated from a soil sample collected at the abandoned Yeonhwa zinc mine located at Gangneung, South Korea. In order to investigate the secondary metabolite production by KMC-002, the strain was cultured in thirty two 1 liter Erlenmeyer flasks each containing 500 mL of A1 liquid medium (total 16 L) [10 g of starch (Difco), 2 g of yeast (Difco) and 4 g of peptone (Difco) in 1 L sterilized distilled water]. Metabolite production in the culture broth was monitored daily by HPLC analysis using an Agilent 1100 LC-MS system with a Phenomenex Luna 5 μm C18(2) analytical column (4.6 × 150 mm, flow rate 0.7 mL/min) and a gradient elution of 10%–100% acetonitrile in water for 30 min. Two major peaks were observed at retention times 28 and 29 min on the fourth day of culture. At the end of the culture period (day 5), sterilized XAD-7 adsorbent resin (20 g/L) was added into the culture broth of the strain KMC-002, and the resin was collected 3 h later by filtration through cheesecloth, washed with deionized water and eluted twice with acetone. The acetone was removed under reduced pressure and the residue was partitioned between ethyl acetate and water. The dried ethyl acetate layer was subjected to a flash column chromatography using reversed phase C18 resin and step gradient elution of water and methanol. The 80% aqueous methanol elution fraction including the major peaks was purified by repeated HPLC using a reversed phase column and gradient elution of acetonitrile and water to afford gombapyrone E (**1**, 5 mg) and F (**2**, 3 mg).

Gombapyrone E (**1**) was isolated as a pale yellowish oil. Its molecular formula was deduced as C_27_H_30_O_3_ on the basis of the HRFABMS (obsd, [M + H]^+^ at *m/z* 403.2278, calcd, 403.2273) and ^13^C-NMR data. The IR spectrum of **1** displayed absorption bands at 3,376 and 1,673 cm^−1^, indicating the presence of hydroxyl and ester carbonyl functionalities, while a UV absorption band at 330 nm was suggestive of a conjugated tetraene-like chromophore [28]. ^1^H-NMR spectral data (Table 1), including correlations from ^1^H-^1^H COSY experiment, illustrated signals attributable to a conjugated tetraene [*δ*_H_ 5.56 (1H, t, *J* = 7.5 Hz), 6.28 (1H, dd, *J* = 15.0, 11.0 Hz), 6.32 (1H, d, *J* = 15.0 Hz), 6.33 (1H, dd, *J* = 11.0, 11.0 Hz), 6.40 (1H, dd, *J* = 15.0, 11.0 Hz), 6.49 (1H, d, *J* = 11.0 Hz) and 6.53 (1H, dd, *J* = 15.0, 11.0 Hz)]. Other characteristic features of the ^1^H-NMR spectrum of **1** were the presence of three additional nonconjugated olefinic protons [*δ*_H_ 4.94 (1H, ddd, *J* = 17.0, 3.5, 2.0 Hz), 4.99 (1H, ddd, *J* = 10.0, 3.5, 2.0 Hz) and 5.90 (1H, ddt, *J* = 17.0, 10.0, 6.5 Hz)], and two methylene protons at *δ*_H_ 3.33 and 3.44. In addition, the ^1^H-NMR spectrum showed additional signals attributed to a phenyl group [*δ*_H_ 7.02 (1H, s), 7.03 (1H, d, *J* = 8.0 Hz) and 7.14 (1H, d, *J* = 8.0 Hz)] and four methyl groups at *δ*_H_ 1.86, 1.91, 1.98 and 2.32. The ^13^C-NMR (Table 1) and HSQC spectra of **1** allowed all protons to be assigned to their respective carbons. The nonconjugated olefinic methylene protons at *δ*_H_ 4.94 and 4.99 correlated with olefinic carbon at *δ*_C_ 114.4, while the nonconjugated olefinic methine proton correlated with a carbon signal at *δ*_C_ 136.9. The four methyl protons at *δ*_H_ 1.86, 1.91, 1.98 and 2.32 correlated with *δ*_C_ 11.3, 7.5, 8.6 and 19.8, respectively, and the methylene protons to carbon signals at *δ*_C_ 37.3 and 29.9. The HSQC spectrum also showed that the phenyl protons at *δ*_H_ 7.02, 7.03 and 7.14 correlated to carbons at *δ*_C_ 129.8, 126.2 and 129.6, respectively. In addition, nine sp_2_ quaternary carbon signals were observed at *δ*_C_ 97.6, 107.9, 133.4, 136.6, 136.8, 138.0, 156.8, 166.5 and 167.0 in the ^13^C-NMR spectrum of **1**.

**Table 1:** ^1^H and ^13^C-NMR spectral data for gombapyrone E (**1**) and F (**2**) in methanol-*d*_4_.

	Gombapyrone E (1)			Gombapyrone F (2)		
Position	*δ*_H_ mult (*J*, Hz) *^a^*	*δ*_C_*^ b^*		*δ*_H_ mult (*J*, Hz)*^ a^*	*δ*_C_*^ b^*	
						
2		167.0	C		166.8	C
3		97.6	C		108.6	C
4		166.5	C		169.3	C
5		107.9	C		110.0	C
6		156.8	C		157.2	C
7	3.44 d (7.5)	29.9	CH_2_	3.46 d (7.5)	29.9	CH_2_
8	5.56 t (7.5)	125.4	CH	5.57 t (7.5)	125.3	CH
9		136.6	C		136.7	C
10	6.32 d (15.0)	137.0	CH	6.33 d (15.0)	137.1	CH
11	6.40 dd (15.0, 11.0)	134.8	CH	6.42 dd (15.0, 11.0)	134.9	CH
12	6.53 dd (15.0, 11.0)	128.9	CH	6.49 dd (15.0, 11.0)	128.9	CH
13	6.28 dd (15.0, 11.0)	128.0	CH	6.32 dd (15.0, 11.0)	128.1	CH
14	6.33 dd (11.0, 11.0)	130.2	CH	6.36 dd (11.0, 11.0)	130.6	CH
15	6.49 d (11.0)	128.3	CH	6.58 d (11.0)	127.9	CH
16		133.4	C		132.5	C
17	7.14 d (8.0)	129.6	CH	7.11 d (8.0)	129.6	CH
18	7.03 d (8.0) *^ c^*	126.2	CH	7.12 d (8.0)	127.3	CH
19		136.8	C		137.0	C
20	7.02 s *^ c^*	129.8	CH	7.32 s	126.6	CH
21		138.0	C		140.8	C
22	3.33 *^d^*	37.3	CH_2_	5.32 d (5.5)	71.2	CH
23	5.90 ddt (17.0, 10.0, 6.5)	136.9	CH	5.96 ddd (17.0, 10.0, 5.5)	139.9	CH
24	4.99 ddd (10.0, 3.5, 2.0)	114.4	CH_2_	5.20 dt (17.0, 1.5)	113.3	CH_2_
	4.94 ddd (17.0, 3.5, 2.0)			5.09 dt (10.0, 1.5)		
25	2.32 s	19.8	CH_3_	2.36 s	19.9	CH_3_
26	1.86 s	11.3	CH_3_	1.86 s	11.3	CH_3_
27	1.98 s	8.6	CH_3_	1.98 s	8.8	CH_3_
28	1.91 s	7.5	CH_3_	2.00 s	8.7	CH_3_
4-OCH_3_				3.87 s	59.7	CH_3_

*^a^* 500 MHz; *^b^* 125 MHz; *^c-^^d^* Overlapping signals; Chemical shifts (*δ*) in ppm.

**Figure 2:**
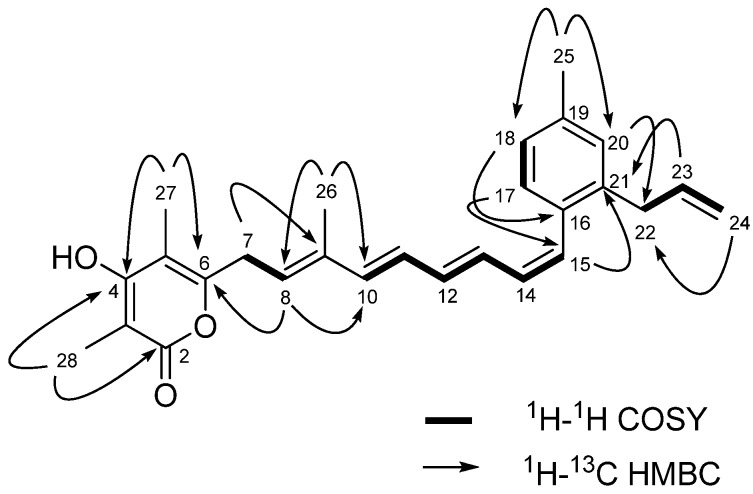
^1^H-^1^H COSY and Key HMBC correlations of gombapyrone E (**1**).

HMBC-NMR correlations from the methyl proton at *δ*_H_ 2.32 (H_3_-25) and the aromatic proton at *δ*_H_ 7.14 (H-17) to the aromatic quaternary carbon at *δ*_C_ 136.8 (C-19) allowed the methyl group to be positioned at C-19. HMBC correlations from the aromatic protons at *δ*_H_ 7.14 (H-17) to the olefinic carbon at *δ*_C_ 128.3 (C-15) allowed the polyene chain to be connected to the C-16 position of the phenyl group. HMBC correlations between the proton signal at *δ*_H_ 5.90 (H-23) and the aromatic quaternary carbon signal at *δ*_C_ 138.0 (C-21) also showed that the allyl functionality from C-22 to C-24 was connected to the C-21 position of the phenyl group (Figure 2). The HMBC correlations from H-27 (*δ*_H_ 1.98) to C-4 (*δ*_C_ 166.5), C-6 (*δ*_C_ 156.8) and C-5 (*δ*_C_ 107.9), and from H-28 (*δ*_H_ 1.91) to C-2 (*δ*_C_ 167.0), C-4 (*δ*_C_ 166.5) and C-3 (*δ*_C_ 97.6) indicated the presence of 3,5-dimethyl-*α*-pyrone. In addition, key HMBC correlations (Figure 2) allowed the polyene chain to be connected to the C-6 position of the *α*-pyrone ring. Comprehensive analysis of 2D-NMR data from ^1^H-^1^H COSY, ^1^H-^1^H TOCSY, NOESY, HSQC and HMBC experiments led to the construction of a 3,5-dimethyl-4-hydroxy-*α*-pyrone and a 2-allyl-4-methyl-benzene connected with the 7-methylnona-1,3,5,7-tetraene chain. The aromatic ring and the *α*-pyrone ring in this specific carbon framework were confirmed by comparison of the proton and carbon NMR chemical shifts of **1 **with those of the previously reported gombapyrone B [26].

**Figure 3:**
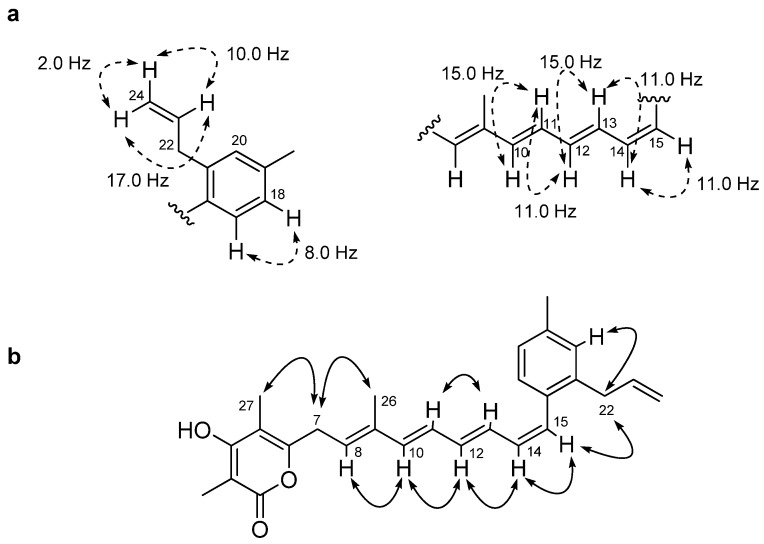
Core ^1^H-^1^H coupling constants (**a**) and Key NOE correlations (**b**) of gombapyrone E (**1**).

The full carbon chain from C-7 to C-15, including the presence of a vinyl methyl group at C-9, was readily assigned by the combined 2D-NMR data of **1**. The configurations of the double bonds in the tetraene chain were assigned on the basis of 1D proton-proton NMR coupling constant data and upon analysis of NOESY-NMR information. The geometries of the Δ^10,11^, Δ^12,13^ and Δ^14,15^ olefins were assigned as *E*, *E* and *Z*, respectively, based upon proton coupling constants of *J*_H-10,H-11_ = 15.0 Hz, *J*_H-12,H-13_ = 15.0 Hz and *J*_H-14,H-15_ = 11.0 Hz (Figure 3). NOESY-NMR correlations between the vinyl methyl proton (H_3_-26) and the H_2_-7 proton, and between H-8 (*δ*_H_ 5.56) and H-10 (*δ*_H_ 6.32) established the configuration of the Δ^8,9^ olefin as *E* (Figure 3).

Gombapyrone F (**2**) was isolated as a pale yellowish oil that analyzed for the molecular formula C_28_H_32_O_4_ by HRFABMS (obsd. [M + Na]^+^ at *m/z* 455.2206, calcd. 455.2198) and by comprehensive analysis of its NMR data (Table 1). The UV absorption spectrum of **2** showed bands at near 330 nm, which were suggestive of the presence of the conjugated polyene as in **1**. The ^1^H-NMR spectrum of **2** was similar to that of **1**, except for methoxy proton and oxygenated methine proton signals at *δ*_H_ 5.32 (1H, d, *J* = 5.5 Hz) and 3.87 (3H, s). The carbon signals observed at *δ* 71.2 and 59.7 supported the presence of these functionalities. The ^1^H-^1^H COSY correlation between the oxygenated methine proton at *δ*_H_ 5.32 (H-22) and H-23 (*δ*_H_ 5.96) and the HMBC correlation from H-23 to C-21 (*δ*_C_ 140.8) easily led to the construction of a 1-hydroxy-allyl group positioned at C-21 of the phenyl group. HMBC-NMR correlations from the methoxy proton to the aromatic quaternary carbon at *δ*_C_ 169.3 (C-4) allowed the methoxy group to be positioned at C-4. The chemical shifts of the *α*-pyrone ring in this specific constellation were comparable to those from gombapyrones as shown in the literature [26]. The double bond geometries of **2** were deduced from ^1^H-^1^H coupling constants and 2D NOE analyses, which were in good accordance with those of **1**.

The gombapyrones E (**1**) and F (**2**) are polyketide secondary metabolites composed of three structural units—*α*-pyrone, tetraene chain and phenyl group. Naturally occurring *α*-pyrone-related compounds have been shown to display a variety of biological activities such as antibiotic, anticancer and neurotoxic effects [29,30,31]. *α*-Pyrones have also attracted great interest due to their effect on HIV protease inhibition and Alzheimer’s amyloid toxicity inhibition [15,32,33,34]. Although the basic carbon skeleton of **1** and **2** have previously been reported by Doi *et al*., a chemical structure with an *α*-pyrone and a phenyl group connected through a tetraene chain is unusual in Nature [27].

The structural composition of *α*-pyrone and phenyl group connected through a linear carbon chain is found in the gombapyrones and lehualides. Previously described gombapyrones, new members of the *α*-pyrone family isolated from *Streptomyces* spp. possess triene chains attached to an aromatic ring, while lehualide A, isolated from marine sponges, is composed of an *α*-pyrone and a benzyl group connected through an eleven carbon chain [4,26].

Compounds **1**-**2** were tested for antimicrobial activity towards six pathogenic microorganisms, including methicillin-resistant *Staphylococcus aureus* (Table 2). Interestingly, **1** showed selective antimicrobial activities against four pathogens (MIC = 3.13 μg/mL against *M. luteus*, 1.56 μg/mL against *S. aureus*, 3.13 μg/mL against *E. hirae* and 6.25 μg/mL against MRSA), whereas **2** did not show any activity at all against all six strains (Table 2). 

**Table 2:** MIC test results for **1** and **2**.

Pathogen list	MIC (μg/mL)		
	1	2	PC *^a^*
*Escherichia coli* KCTC 2593	NA	NA	6.25
*Micrococcus luteus* KCCM 11548	3.13	100	0.78
*Staphylococcus aureus* KCTC 1916	1.56	NA	0.78
*Bacillus subtilis* KCTC1021	NA	NA	3.13
*Enterococcus hirae* KCCM 11768	3.13	100	0.78
*Staphylococcus aureus* MRSA 2659	6.25	NA	12.5

*^a ^*Positive control (PC) = Ampicillin; NA = Not Activity.

## 3. Experimental

### 3.1. General

A Waters 1525 system with a Phenomenex Luna C18 (2) 5 *μ*m column (4.6 × 100 mm) and a PDA detector was used for HPLC analysis. Low resolution MS data was measured on an Agilent 1100 LC/MS system equipped with a Phenomenex Luna C18 (2) 5 μm column (4.6 × 150 mm, flow rate 0.7 mL/min). Lichroprep RP-18 (Merck, 40–63 μm) was used for reversed phase flash column chromatography. HPLC separation and purification were performed using a Gilson 321 HPLC system with a Phenomenex Luna C18 (2) 10 μm column (21.2 × 250 mm) at a flow rate 10 mL/min or Phenomenex Luna C18 (2) 10 μm column (10 × 250 mm) at a flow rate 4 mL/min. All NMR spectra were obtained in methanol-*d*_4_ (*δ*_H_ 3.32, *δ*_C_ 47.6) on a Varian UNITY Plus 500 MHz NMR system equipped with a cryogenic probe. FT-IR spectra were recorded on a Thermo Scientific Nicolet iS10 spectrometer. UV spectra were obtained on a PerkinElmer Lambda 35 UV/Vis spectrophotometer. Optical rotations were measured on a PerkinElmer model 343 polarimeter. HRFABMS data were obtained on a JEOL/JMS-AX505WA instrument.

### 3.2. Strain Collection, Isolation, and Identification

Soil samples were collected at the abandoned Yeonhwa zinc mine located at Gangneung, South Korea. The samples were diluted with autoclaved distilled water, heated to 55 °C for 6 min and the resulting suspension (100 μL) inoculated onto several kinds of agar plate. The plates were incubated 2 weeks at 25 °C under aerobic conditions. A pure *Streptomyces* strain KMC-002 was isolated from A1 agar medium (10 g of starch, 4 g of yeast, 2 g of peptone and 18 g of agar in 1 L of sterile distilled water). Stocks of the isolated bacterial strain were generated and stored at −80 °C in liquid culture medium containing 15% (v/v) glycerol. The strain KMC-002 was identified based on 16S rRNA gene sequencing analysis. The chromosomal DNA of strain KMC-002 was extracted using the G-spin genomic DNA extraction kit (iNtRON Biotechnology, Daejeon, Korea). The 16S rRNA gene of strain KMC-002 was amplified by PCR using universal primers 27f and 1492r corresponding to positions 27 in the forward direction and 1492 in the reverse direction of the *Escherichia coli* 16S rRNA gene [35]. The DNA sequencing reaction was carried out by using an ABI Prism Bigdye terminator cycle sequencing ready reaction kit V.3.1 (Applied Biosystems, Foster City, CA, USA). The PCR cycle-sequencing product was purified by using Montage dye remove kit (Millipore, Bedford, TX, USA) according to the manufacturer’s protocol. 16S rRNA gene sequence was determined on a Perkin-Elmer model ABI 3730XL capillary DNA sequencer (Applied Biosystems). The 16S rRNA gene sequence of strain KMC-002 was compared with primary sequence information within the GenBank/EMBL/DDBJ nucleotide sequence database using the BLAST algorithm [36].

The strain KMC-002 was classified as a *Streptomyces* sp. based on 16S rDNA sequence analysis which showed 99.1% similarity with *Streptomyces albireticuli* strain JCM 4116 (AY999748). The cultured *Streptomyces* strain KMC-002 was deposited with the Korean Culture Center of Microorganisms (KCCM 90098). 

### 3.3. Cultivation and Extraction

The strain KMC-002 was cultured in 1 L Erlenmeyer flasks (32 × 500 mL, total 16 L), each containing 500 mL of A1 liquid medium (10 g of starch, 4 g of yeast, 2 g of peptone and 1 L of sterile distilled water) while shaking 200 rpm at 27 °C. After 5 days, sterilized XAD-7 adsorbent resin (20 g/L) was added into the culture broth of the strain KMC-002, and the resin was collected 3 h later by filtration through cheesecloth, washed with deionized water and eluted twice with acetone. The acetone was removed under reduced pressure and the residue was partitioned between ethyl acetate and water. The ethyl acetate layer was dried *in vacuo* to yield 800 mg of crude extract.

### 3.4. Isolation and Purification of Gombapyrones E (1) and F (2)

The crude extract was fractionated by Lichroprep RP-18 (40–63 μm, Merck, N.J., USA) flash column chromatography using a step gradient elution with H_2_O and CH_3_OH (20%, 40%, 60%, 80%, and 100% CH_3_OH in H_2_O) to give six subfractions. The 80% mathanol fraction contained gombapyrone E(**1**) and F(**2**), which were separated by reversed phase HPLC [Gilson 321; Phenomenex Luna C18 (2) 10 μm column (21.2 × 250 mm); 10 mL/min] using gradient elution from 10% to 100% aqueous CH_3_CN containing 0.02% trifluoroacetic acid (TFA) for 2 h. **1** (5 mg, *t*_R_ 50 min) and **2** (3 mg, *t*_R_ 40 min) were purified by reversed phase HPLC [Gilson 321; Phenomenex Luna C8 (2) 10 μm column (10 × 250 mm), 4 mL/min] with an CH_3_CN-H_2_O gradient elution system from 50% CH_3_CN to 100% CH_3_CN over 1 h.

*Gombapyrone E* (**1**): Pale yellowish oil; 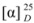: −3.2 (*c* 0.10, MeOH); UV (MeCN) *λ*_max_ (log *ε*) 200 (4.27), 260 (3.70), 329 (4.28); IR (film) *ν*_max_ 3,376, 2,925, 1,672, 1,378, 1,202, 756 cm^−1^; ^1^H- and ^13^C-NMR spectra: see Table 1; HRFABMS [M + H]^+^ *m/z* 403.2278 (calcd. for C_27_H_31_O_3_, 403.2273).

*Gombapyrone F* (**2**): Pale yellowish oil; 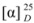: −6.0 (*c* 0.05, MeOH); UV (MeCN) *λ*_max_ (log *ε*) 200 (4.07), 260 (3.52), 332 (4.06); IR (film) *ν*_max_ 3,356, 2,925, 1,683, 1,456, 1,026, 821 cm^−1^; ^1^H- and ^13^C-NMR spectra: see Table 1; HRFABMS [M + Na]^+^ *m/z* 455.2206 (calcd. for C_28_H_32_O_4_Na, 455.2198).

### 3.5. Antibacterial Activities Test

The antibacterial activities of **1** and **2** were evaluated by MIC assay against six pathogenic microorganisms, *Escherichia coli* (KCTC 2593), *Bacillus subtilis* (KCTC 1021), *Staphylococcus aureus* (KCTC 1916), Methicillin resistance *Staphylococcus aureus* MRSA 2659, *Micrococcus luteus* (KCCM 11548) and *Enterococcus hirae* (KCCM 11768). For MIC assays, all six strains were grown at 37 ºC in nutrient agar (Difco, USA). A loopful of all strains was resuspended in nutrient broth to give 10^5^ colony forming units/mL and incubated at 37 ºC for 18 h and dispensed into 96 well plates (100 μL/well). The MIC evaluation was determined as the concentration of the compounds that completely inhibited pathogenic cell growth for 24 h incubation at 37 ºC. Ampicillin was used as a positive control.

## 4. Conclusions

Two new *α*-pyrone polyenes, gombapyrones E (**1**) and F (**2**), were isolated from the abandoned mine-derived *Streptomyces* sp. KMC-002. The key structural features of **1** and **2** were characterized by spectroscopic methods, including 2D-NMR experiments, as a 3,5-dimethyl-4-hydroxy-*α*-pyrone and a 2-allyl-4-methylbenzene connected by a 7-methylnona-1,3,5,7-tetraene chain. Gombapyrone E showed antibacterial activity against *Micrococcus luteus*, *Enterococcus hirae*, *Staphylococcus aureus* and MRSA. For decades, investigation at the chemical and biochemical levels to better understand polyketide diversity has been continuously making impressive progress in the natural products field. Isolation of gombapyrones E (**1**) and F (**2**) is expected to contribute towards providing a better understanding of the structural diversity of polyketide natural products.
